# 

**Published:** 1877-12

**Authors:** 


### Contents of December Number.

### ORIGINAL.

ART. I.—Materia Medica and New Remedies. By Thomas D. Washburn, M. D., .......... 161
ART. II.—External Glandular Enlargements. By Z. Collins McElroy, M. D., .......... 168
ART. III.—Summary of Remarks on Fractures of the Shaft of the Femur in Adults. By Prof. Frank H. Hamilton. Reported by J. C. Lay, M. D., M. R. C. S. E., .......... 173

### CORRESPONDENCE.

Medical Advertisements through Dispensaries, etc., .......... 175

### MISCELLANEOUS.

The Abuse of Medical Charities, .......... 177
Ferrum Dialysatum, .......... 182
Typhoid Infection, .......... 183
Nature of Communicable Diseases, .......... 184
A New Instrument for the Administration of Compressed Air, .......... 185
Diphtheria, Croup and Tracheotomy, .......... 186
Ovariotomy during Pregnancy, .......... 187
Pepeating Physicians Prescriptions, .......... 187

### OBITUARY

Prof. Paul E. Eve, M. D., .......... 187
Prof. Geo. Hadley, .......... 188

### EDITORIAL.

Modern Homeopathy, .......... 192
Lectures in the Buffalo Medical College, .......... 193
BOOKS REVIEWED:
Forensic Medicine and Toxicology. Woodman and Tidy, .......... 194
Lectures on Practical Surgery. Toland, .......... 194
The Ear. Burnett, .......... 195
Wood's Vade-Mecum, .......... 196
The Practitioner's Reference Book. Dunglison. An Index of Diseases and their Treatment. Tanner, .......... 196
Biology. Cook, .......... 198
Books and Pamphlets Received, .......... 199
List of Exchanges, .......... 200

---

THE BUFFALO

